# Meeting of the British Medical Association, Toronto

**Published:** 1906-08-25

**Authors:** 


### Meeting of the British Medical Association, Toronto.

### THE ADDRESSES.

### ADDRESS IN OBSTETRICS.

#### The Teaching of Obstetrics.

At the annual meeting of the British Medical Association at Toronto the address on Obstetrics was delivered by Dr. W. S. A. Griffith, Physician Accoucheur, St. Bartholomew's Hospital, London.

He reviewed the history of gynæcology as revealed in the various addresses delivered at previous meetings of the British Medical Association, and dealt with the teaching and training of the medical students in practical obstetrics. He mentioned the great difficulty of providing practical clinical instruction at the four lying-in hospital charities in London where the instruction was directed to the training of monthly nurses and midwives, with the exception of Queen Charlotte's Hospital. At the present time it was attended chiefly by qualified men, who, having found out their lack of experience in this subject, were glad to make use of the opportunities for instruction to be obtained in that institution. Externe maternity departments attached to various medical schools were under more or less organised supervision, and, when attended by students who had the necessary preliminary clinical teaching, provided a most important and valuable source of training. The opinion which held sway in England—that the process of labour was a simple function of nature—has led to the study of obstetrics being relegated to a position totally unworthy of its immense importance to the practitioner of obstetrics and to the national welfare. Another defect in the present teaching system was due to the fact that frequently the lecturers had little experience in the practice of obstetrics, attending sometimes not more than twenty cases in the year, and limiting their experience to a few special cases. In many of the provincial schools the lecturer was a successful general practitioner, to whom the fates had given a large obstetric experience, but whose scientific training and experience as a teacher was inadequate for this work. Dr. Griffith recommended that special hospitals should be provided for the purpose of clinical teaching in obstetrics with a large externe department connected with each. He was convinced that it would be of great advantage to teachers and pupils that medical students be taught their duties at the same time and place as monthly nurses. Owing to the great difficulty in providing the necessary funds for the maintenance and development of existing hospitals, it seemed hardly possible to expect adequate accommodation for the teaching of obstetrics in the near future unless such hospitals were placed on the same basis as the splendid provision made for patients in the great public asylums and fever hospitals. He was inclined to look forward to a time when the great hospitals of the country would be relieved from the eternal difficulty of finding funds and when they would be equipped in an equally suitable, though perhaps less expensive, manner by funds provided by the State, administrated by the existing Board of Managers, under the general supervision of a responsible Central Hospital Board or Commission.

The remainder of the lecture was devoted to the recommendations on the subject of obstetric teaching by the General Medical Council, and to a eulogy of the work of the late Dr. Matthews Duncan, whose influence, personal qualities, knowledge, experience, and clear judgment had raised the standard of the science and practice of obstetrics to a position which, at least in London, they had never before attained.

### ADDRESS IN MEDICINE.

#### The Circulation viewed from the Periphery.

The address in medicine was delivered by Sir James Barr, Senior Physician to the Liverpool Royal Infirmary. He dealt with the subject of the circulation of the blood in great detail, placing most stress upon the physiology and physics of the circulation. Diseases of the heart, he said, most frequently arose from causes acting upon the periphery, and the man who studied the circulation only with the aid of a stethoscope was a positive danger to society. After describing the capillaries, he said that in very plethoric individuals and in cases of polycythæmia the capillaries of the body were fairly replete, but in ordinary mortals, especially in those of neurotic temperament, perhaps not a third of the capillaries were full at any one moment. This had a very great influence on the pressure and velocity of the capillary circulation. Anyone with a capillary velocity at the level of the heart, which physiologists set down as normal, might, he said, appropriately take up the refrain, "The hour of my departure's come." He had corroborated v. Kries's observations as to the effects of gravity on capillary pressure, and, like him, had found that the increase was usually less than one-half the hydrostatic effect. He had found that the increase in response to gravitation varied enormously in the same individual under different conditions, and largely depended on the condition of the vasomotor mechanism of the part examined. He had shown that the capillary pressure in the foot, even when immobilised, was less than that in the hand, and much less than that in a grog-blossomed nose. This was entirely due to the wonderful mechanism of the vasomotor system.

Some people were very liable to cold feet in bed, and such appendages to a lady seem to have led up to a divorce in the United States of America. In such cases the part may be fairly comfortable before going to bed, but once the horizontal posture is assumed the arterial pressure and capillary velocity fall, there is not a sufficient amount of fuel carried to the extremities to keep the large cooling surface warm. Here the defect is in the initial energy, and besides improving the general arterial pressure, it would be advantageous to keep the feet much lower than the head and shoulders.

The capillary velocity varied enormously in different individuals under different conditions, and depended chiefly on the potential in the arteries—the higher the arterial pressure, the greater the velocity in the arterioles and capillaries. Obstructions to the outflow from the capillaries diminishes the velocity in them.

When the velocity in the capillaries was reduced to one millimetre or less per second the blood becomes surcharged with carbonic acid, and the skin or organ supplied becomes of a dusky hue. This appearance immediately disappears if you increase the capillary velocity; for example, when the hand is blue and passively congested from cold, or the so-called local asphyxia, if you let it hang down you increase the velocity, and you quickly see bright red spots intermingled with surrounding lividity, and soon the colour of the whole hand improves. In the cold livid dependent hand the colour of the fingers is better than that of the back of the hand. When cardiac failure occurs, with or without any obstructive lung disease, the upper part of the body and the hands are quite dusky, while the legs and feet, which are at a lower level, may be pale. In one marked case of cardiac failure where the upper part of the body was livid, I saw one foot and part of the leg in a state of local syncope and as pale as marble. These patients do not require a cylinder of oxygen, with which they are frequently plied, but the judicious application of a little common sense, such as the intravenous injection of small doses of adrenalin or some cardiac tonic. In cases of Raynaud's disease the local syncope is ascribed to vasomotor spasm, but really the spasm, if it exist, is a very mild affair. In these cases the arterioles shut down because there is not sufficient blood pressure to keep them open. The arterial pressure is always low, and the blood is deficient in lime salts and viscosity. In the cases of local asphyxia the arterioles are not closed, but the arterial potential is low, the velocity in the capillaries is defective, and the *vis viva* is not sufficient to drive on the blood stagnating in the veins. In cases of erythromelalgia the reverse happens; the velocity and pressure are both increased in the large engorged capillaries.

In many cases of pneumonia with low blood pressure, the vasomotor taps in the splanchnic area are all open, and the aorta is drained before it terminates in the iliac arteries; the bulk of the blood is retained in the chest and abdomen, and the quantity supplied to the lower limbs is diminished. Moreover, the extremities are often colder than the body, and the arteries contracted. The lower level of the limbs increases the velocity in the capillaries and veins, and consequently the capillaries of the foot and leg are often blanched and the veins comparatively empty when the upper part of the body appears congested and purple.

The remaining part of the lecture dealt with the viscosity of the blood, and mentioned the work of Professor Sir A. E. Wright as showing that viscosity was increased by the salts of calcium, magnesium, and strontium, and diminished by decalcifying agents such as citric acid and the salts of potassium, ammonium, and sodium.

Sir James Barr dealt with the action of the arterioles and capillaries of the skin, splanchnic area, liver, kidneys, muscles, cerebral vessels, coronary vessels, and with the various phenomena of the pulmonary and venous circulations, and concluded by giving an account of the physiology of the arteries and the heart: all tending to show the necessity of a well-balanced circulation for the maintenance of life and health.

---

### ADDRESS IN SURGERY.

### On the Technique of Operations on the Central Nervous System.

The Address in Surgery was delivered on Wednesday, August 22, by Sir Victor Horsley, F.R.C.S., F.R.S., Surgeon to University College Hospital, in the course of which he proceeded to analyse the cases, demonstrating certain points of technique derived from his experience at the National Hospital, Queen Square, at University College Hospital, and in private practice. Correct diagnosis in diseases of the nervous system were still far to seek, and yet good results had been obtained. Vital pathology and the true anatomical nature of brain diseases had been demonstrated in the operating theatre. Post-mortem records could never teach what a careful study of the living tumours exposed in an operation could demonstrate. So long as the powers of diagnosis remain as imperfect as they are, so long will the vulgar error of regarding surgical treatment as the last resource in such cases be committed. It had been proposed, for example, that Jacksonian epilepsy, to have a definite probationary period of medicinal treatment agreed upon, and that in an elementary case where no urgent symptoms like optic neuritis existed, surgical treatment should be employed after thorough drug medication had lasted for about six or eight weeks.

Then came the question for what precise purpose was the operation to be employed, whether palliative or curative. Sir Victor considered the details of operative procedure, including previous preparation of the patient and anæsthesia, regarding which he said that intraspinal anæsthesia is of too limited applicability, because control of the intensity of narcosis was lost, but he suggested a combined anæsthesia of morphine with chloroform, but had given it up and employed pure chloroform only for many years. He regarded ether as inadmissible as an anæsthetic in operations on the nervous system.

Chloroform, *per contra*, causes a fall of blood-pressure with relatively less blood venosity—although this is by no means absent, as will be seen below. It therefore does not aggravate the bleeding, nor embarrass the respiration by causing bronchorrhœa.

By its more essentially paralysing action on nerve centres it causes practically no after-excitement and but moderate headache. It is probably as frequently followed by obstinate sickness, but this depends on many other correlative factors, and primarily on the dose used (*vide infra*). Chloroform, however, as already stated, is more dangerous. It kills by paralysis of the respiratory centre as often or more often than by paralysis of the heart.

Moreover, all cases of increased intercranial tension (as is now well recognised) are liable to die at any moment from sudden paralysis of the respiratory centre. How often one sees this accident in cases of intercranial tumour which are only at the very last transferred for surgical treatment.

Deaths had occurred on the operation-table in cases where tumour pressure had hampered the bulb, and chloroform, therefore, had to be used with caution in the surgery of the nervous system to avoid giving a dose which might bring about fatal arrest of the respiratory centre. The immediate problem was how to regulate dosage of chloroform. The Research Committee of the Association had found that less than 2 per cent. of chloroform vapour in the atmosphere breathed by a patient is enough to produce deep narcosis, and that a much smaller dose is required to maintain unconsciousness.

If the mask of the inhaler be made to fit by wet aseptic towels, the amount of the dose given will be under complete control. With the dose commencing at 0.5 per cent., and rising in one to two minutes to 2 per cent., the patient ought to be ready for operation after five to eight minutes. If the initial narcosis be complete no adverse event—for example, vomiting—will occur. If it be incomplete when the operation is commenced various drawbacks will appear. This is, of course, well recognised as a general principle by anæsthetists, but is so salient a point as to deserve repetition.

As a rule the amount of 2 per cent. is given for about five minutes before the incision of the skin and reflection of the flap which constitutes the maximal pain period of the operation. This completed, the dose can be lowered by pushing the tap back and the bone be removed at 1 per cent. As the dura is a sensitive membrane supplied by the fifth cranial nerve the dose should be somewhat raised just previous to its incision to prevent reflex starts or movements on the part of the patient.

As soon as the dura is opened the encephalon can be dealt with without causing any pain except if the course of the fillet or one of the peripheral sensory cranial nerves be accidentally irritated. Consequently all this part of the operation is done under less than 0.5 per cent. of chloroform in the air respired, an amount which, of course, is far below that required in the induction stage.

Dealing with the necessity for maintaining the body-temperature, Sir Victor pointed out that general anæsthesia possessed a high degree of power to lower the temperature of the body, and therewith emphasise the shock of the operation.

This is characteristic of all narcotic substances (*cf.* Chloral, Lauder Brunton). Ether, for example, in a very short time will, as Dr. Hare has shown, lower the temperature of the body two degrees Fahrenheit. For this reason all operating-rooms should be at a temperature of not less than 75° F., and that the operating-table should be provided with a suitable hot-water bed.

While, however, cooling due to the anæsthesia can thus be readily combated, it is necessary during all operative procedures on the skull and its cavity to prevent cooling by radiation from the brain exposed in the wound. The wound, therefore, should be constantly irrigated, usually with a solution of sublimate of 1 in 10,000 strength, or with saline. These lotions are put into the irrigator at a temperature of 115° for the reason to be detailed directly, and the flow is regulated at will by an assistant.

The use of the hot irrigation fluid, however, is not only to prevent cooling of the nerve-centres; it also has another purpose—namely, the arrest of capillary and arterial hæmorrhage.

In dealing with hæmorrhage the first general principle is the recognition of the fact, originally established by Cohnheim's researches, that as few vessels as possible should be obstructed; and this principle must be followed as closely in the case of the veins as in the case of arteries. In pursuance of this principle, where it is necessary to remove large portions of the brain, the branches of vessels to be divided should be severed as far as possible from the trunk.

Sir Victor dealt with the subjects of hæmorrhage from arteries, arterio-capillaries, oozing, shock and its treatment. He concluded by saying that the following deductions were justified:—

(1) That operation should be resorted to as early as possible; (2) the tumour should be, if possible, freely exposed and examined and extirpated with surrounding tissue; (3) that if it cannot be removed without undue interference with important or essential structures there remains some possibility of the tumour undergoing retrogression in a certain number of cases.

